# Identification of Prognostic and Therapeutic Biomarkers among FAM83 Family Members for Pancreatic Ductal Adenocarcinoma

**DOI:** 10.1155/2021/6682697

**Published:** 2021-03-01

**Authors:** Zuyi Ma, Zixuan Zhou, Hongkai Zhuang, Zhenchong Li, Zuguang Ma, Bowen Huang, Chunsheng Liu, Yuanfeng Gong, Yiping Zou, Zehao Zheng, Shanzhou Huang, Chuanzhao Zhang, Baohua Hou

**Affiliations:** ^1^Department of General Surgery, Guangdong Provincial People's Hospital, School of Medicine, South China University of Technology, Guangzhou 510080, China; ^2^Shantou University of Medical College, Shantou 515000, China; ^3^Sanshui Disease Prevention Cure Station, Foshan 528100, China; ^4^Department of General Surgery, Peking Union Medical College Hospital, Chinese Academy of Medical Sciences and Peking Union Medical College, Beijing 100730, China

## Abstract

Family with sequence similarity 83 (FAM83) members were shown recently to have oncogenic effect in a variety of cancer types, but the biological roles and prognostic value of FAM83 family in pancreatic ductal adenocarcinoma remain unknown. In the current study, the clinical significance and molecular function of the FAM83 family were assessed by multiple bioinformatics analysis. Besides, potential associations between differentially expressed genes (DEGs) of FAM83 family and antitumor immunity were evaluated using TIMER and TISIDB analyses. As the results show, FAM83A, FAM83D, FAM83E, and FAM83H were significantly upregulated in PDAC and were identified as DEGs. Higher expression of FAM83A, FAM83B, FAM83D, FAM83E, and FAM83H were associated with advanced tumor stage or worse patient prognosis. Importantly, the overexpression of DEGs was found to be significantly correlated with activated KRAS and loss of SMAD4, which are important drivers for PDAC. Further, FAM83A, FAM83D, and FAM83H were associated with CD8^+^ T cell, Gamma Delta T cell, and CD4^+^ T cell infiltration in PDAC and FAM83H was found closely correlated with some immunomodulators including immunoinhibitors, immunostimulators, and MHC molecules. In conclusion, FAM83A, FAM83D, FAM83E, and FAM83H have significant prognostic value in PDAC and they may play important roles in regulating tumor progression and the immune cell infiltration.

## 1. Introduction

Pancreatic cancer (PC) is considered to be one of the most aggressive cancers, leading to 4.7% of all cancer-related deaths globally [1]. Pancreatic ductal adenocarcinoma (PDAC) accounts for more than 80% of PC cases, with approximately 10% of surviving rate beyond five years [2]. One of the vital causes for the poor prognosis is the highly aggressive phenotype and early recurrence and metastasis of PDAC following surgical treatment [3, 4]. Recently immune checkpoint inhibitors have been widely used in several solid tumors including hepatocellular carcinoma, non-small-cell lung cancer, and melanoma [5–7]. However, PC was considered a “cold” tumor and exhibited limited efficacy due to its notable immunosuppression [8]. Therefore, there is an urgent need to explore the molecular mechanisms underlying PC progression and immune suppression as well as to identify early diagnostic, prognostic, and therapeutic biomarkers for PDAC.

In recent studies, some of the family with sequence similarity 83 (FAM83) family members have been demonstrated significantly upregulated in a variety of human cancer types [9]. There are eight FAM83 family members, named FAM83A-H, with each located at a distinct genomic site. Each FAM83 gene encodes a protein classified solely on the presence of a highly conserved domain of unknown function (DUF1669) located in the N-terminus [10]. However, each member has a unique C terminus of variable length and their biological function and related mechanism may be distinct. Accumulating evidence also demonstrated significant roles for some FAM83 family members in tumorigenesis and tumor progression [9]. Expressions of FAM83A and B in breast cancer were found involved in the PI3K and EGFR pathway, making surviving tumor cells resistant to TKI therapy [11, 12]. FAM83D have been identified as potential key regulators in cell invasion and proliferation of ovarian cancer, which also inhibited autophagy via the PI3K/AKT/mTOR signaling pathway [13]. However, the biological role of FAM83 family members in PDAC remains unclear, while a detailed understanding of biological and molecular mechanism is critical to develop novel treatment options.

In the current study, we first analyzed the transcriptional levels of FAM83 family members in PDAC. Then, we assessed the associations between FAM83 family expression with pathological stage or patient survival to evaluate the value of the FAM83 family in the progression and prognosis of PDAC. Differentially expressed genes (DEGs) in FAM83 family members of PDAC were integrated to DAVID 6.7 to perform functional enrichment analyses. Further, we explored the biological roles of FAM83 family members in the immune infiltration of PDAC.

## 2. Materials and Methods

### 2.1. Oncomine Database Analysis

Oncomine (https://www.oncomine.org) is a publicly accessible online bioinformatics database that contains 715 datasets, as well as 86,733 normal and tumor samples, and provides powerful genome-wide expression analysis [14]. The mRNA levels of FAM83 family members were analyzed in PDAC and a fold change of 1.5 and a *p* value of 0.05 were set as the significance thresholds. Student's *t*-test was used to evaluate the difference of FAM83 family members in PDAC.

### 2.2. GEPIA Database Analysis

Gene Expression Profiling Interactive Analysis (GEPIA) (http://gepia.cancer-pku.cn/index.html) is a web-based tool for analyzing transcriptional data of 9,736 tumors and 8,587 normal samples from The Cancer Genome Atlas (TCGA) and the Genotype-Tissue Expression (GTEx) projects [15]. GEPIA provides customizable functions such as tumor/normal differential expression analysis, profiling according to cancer types or pathological stages, patient survival analysis, similar gene detection, correlation analysis, and dimensionality reduction analysis. In our study, the database was used to validate differential transcriptional levels in PDAC and normal tissues, as well as pathological stage analysis, relative expression analysis, and correlative prognostic analysis. Student's *t*-test was used to analyze the expressions or pathological stages. The fold change cutoff was 1.5, and the *p* value cutoff was 0.05. Prognostic analysis was performed using a Kaplan-Meier curve.

### 2.3. Gene Correlation Analysis

The transcriptional expression data based on fragments Per kilobase per million (FPKM) for PDAC were obtained from TCGA database (https://cancergenome.nih.gov/). Of the 177 PDAC cases obtained, the correlation of mRNA expression levels among FAM83 family was evaluated by using R software (version 3.5.3) with “corrplot” package.

### 2.4. cBioPortal Database Analysis

cBioPortal Database (http://www.cbioportal.org/) is a web resource for visualizing and analyzing a wide variety of cancer genomics data retrieved from TCGA database [16]. It lowers the barriers between complex genomic data and cancer researchers by providing rapid, intuitive, and high-quality access to molecular profiles and clinical attributes from large-scale cancer genomics projects and therefore empowers researchers to translate these rich data sets into biologic insights and clinical applications. In the current study, the cBioPortal database was used to evaluate genetic mutations among some FAM83 family members in PDAC and evaluate their correlations with genes related to PDAC (e.g., KRAS). In addition, coexpression genes of DEGs in FAM83 family members were screened out through calculating the Spearman correlation coefficients, respectively (Spearman's correlated coefficient > 0.6 or <−0.6, *p* value < 0.05).

### 2.5. Functional and Pathway Enrichment Analysis

Coexpressed genes screened from cBioPortal database were integrated to DAVID 6.7 (https://david-d.ncifcrf.gov/) to perform Gene Ontology (GO) analysis and Kyoto Encyclopedia of Genes and Genomes (KEGG) pathway analysis [17]. Results were visualized by using R software (version 3.5.3) with “ggplot2” package, and *p* value < 0.05 was considered statistically significant.

### 2.6. GeneMANIA Database Analysis

GeneMANIA database (http://genemania.org/) is a user-friendly website for exploring internal relationships of gene sets [18, 19]. The gene interaction network for FAM83 family members was constructed by using GeneMANIA.

### 2.7. TIMER 2.0 Database Analysis

TIMER 2.0 (http://timer.cistrome.org/) is a webserver for systematical analysis of immune infiltration across a wild variety of cancer types [20]. The webserver provides immune infiltrates' abundances estimated by multiple immune deconvolution methods and allows users to generate high-quality figures dynamically to explore tumor immunological, clinical, and genomic features comprehensively. This online tool was used to evaluate the correlations of FAM83 family members with immune cell infiltration levels including CD8^+^ T cell, Gamma Delta T cell, follicular helper T cell, and CD4^+^ T cell. Spearman's correlation coefficients were used, and *p* values < 0.05 were considered statistically significant.

### 2.8. TISIDB Database Analysis

TISIDB (http://cis.hku.hk/TISIDB) is an integrated repository web for tumor immunity, through which biologists can crosscheck a gene about its role in tumor-immune interactions through literature mining and high-throughput data analysis [21]. In this study, the TISIDB platform was used to analyze correlations between FAM83H expressions with tumor-infiltrating lymphocytes (TILs) and immunomodulators (including immunostimulators, immunoinhibitors, and major histocompatibility complex (MHC) molecules). Spearman's correlation coefficients were used, and *p* values < 0.05 were considered statistically significant.

## 3. Results

### 3.1. Differentially mRNA Expression Levels of FAM83 Family in PDAC

**Figure 1:**
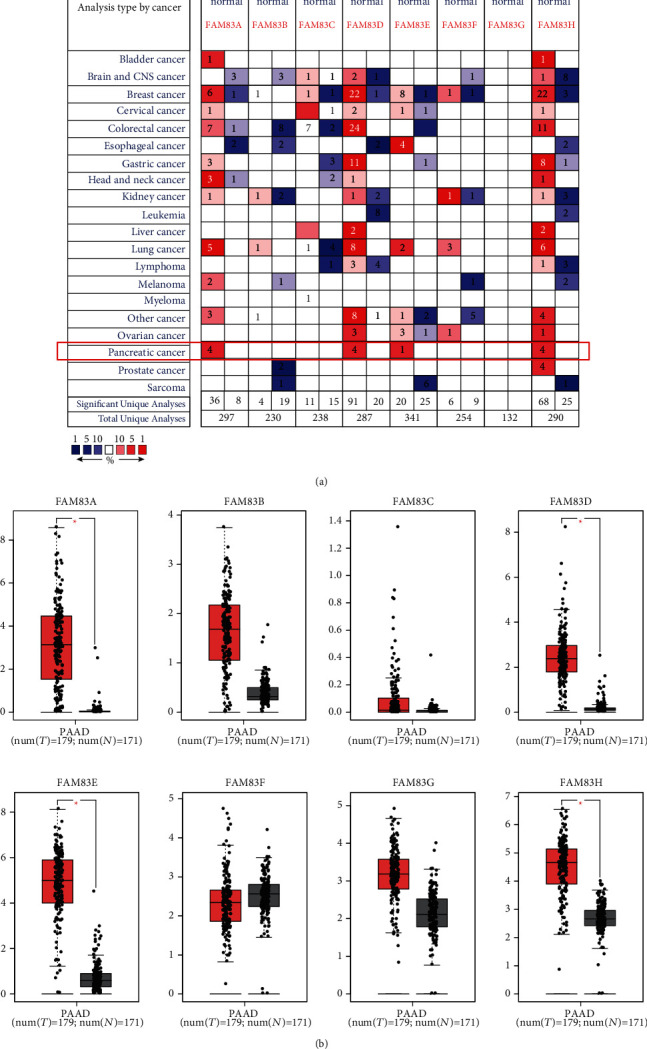
Transcriptional levels of FAM83 family members in pancreatic ductal adenocarcinoma (PDAC). (a) Oncomine dataset analysis showed the numbers of datasets with significant transcriptional upregulated expression (red) or downregulated expression (blue). (b) GEPIA dataset analysis validated increased FAM83A, FAM83D, FAM83E, and FAM83H expression in PDAC tumor. The fold change cutoff was 1.5, and the *p* value cutoff was 0.05. Transcriptional expression levels of FAM83 family members in PDAC are delineated with red highlights.

Eight FAM83 family members were analyzed using the Oncomine database in various cancer types, and the results showed that there were a total of 297, 230, 238, 287, 341, 254, 132, and 290 unique analyses for FAM83A, FAM83B, FAM83C, FAM83D, FAM83E, FAM83F, FAM83G, and FAM83H, respectively. Based on the data from Oncomine, the transcriptional levels of FAM83A, FAM83D, FAM83E, and FAM83H were significantly upregulated in PDAC tissues compared to normal pancreas tissues (Figure 1(a)). In the analysis by Iacobuzio-Donahue, the mRNA level of FAM83A (*p* value = 7.60*e*-04) was significantly increased with a fold change of 2.838 in PDAC [22]. Pei et al.'s dataset suggested that FAM83A had a fold induction of 2.681 and a *p* value of 2.41*e*-07 [23]. Similar results were reported by Grützmann et al. (fold change = 2.209 and *p* value = 0.047) and Badea et al. (fold change = 1.557 and *p* value = 3.21*e*-05) in PDAC [24, 25]. As shown in Table 1, these 4 unique analyses also showed a significant elevation of FAM83D and FAM83H expression in PDAC. And the FAM83E expression was shown to be higher in PDAC in one dataset (fold change = 2.365 and *p* value = 3.07*e*-06) [23]. In addition, we performed the analysis in another independent dataset, the GEPIA dataset, and validated that the expression levels of FAM83A, FAM83D, FAM83E, and FAM83H were increased in PDAC tumor compared with normal samples (Figure 1(b)). Taking these together, by bioinformatics analysis using data from Oncomine and GEPIA database, FAM83A, FAM83D, FAM83E, and FAM83H were found to be upregulated in PDAC.

### 3.2. Relative Expression, Coexpression, Genetic Alteration, and Neighbor Gene Network of FAM83 Family in PDAC

**Figure 2:**
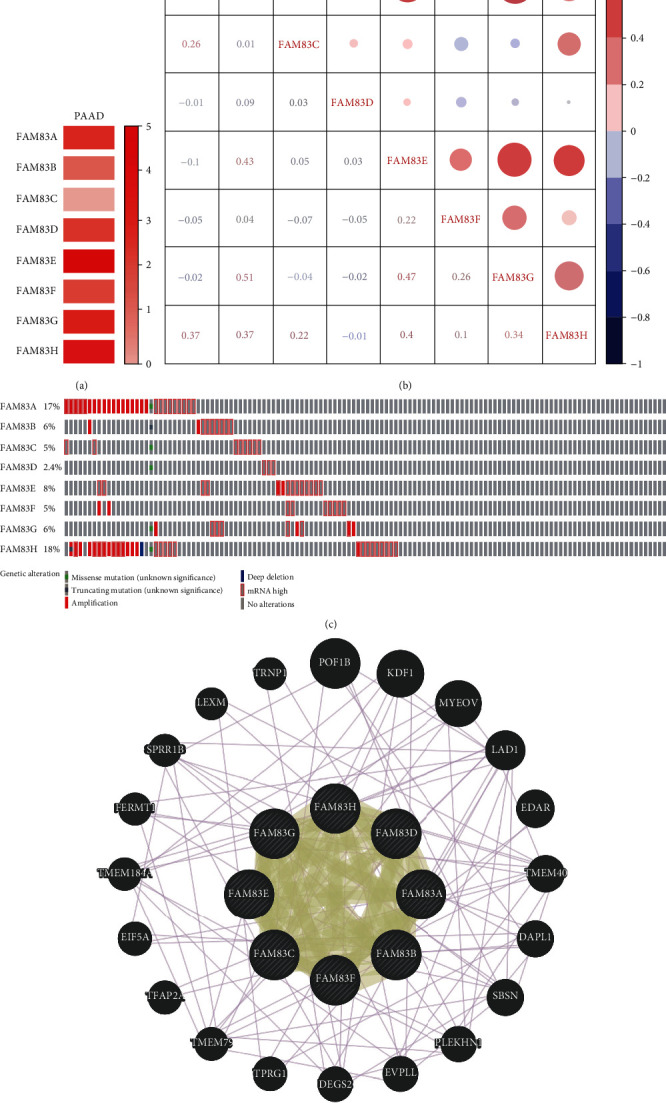
Relative expression, coexpression, genetic alteration, and neighbor gene network of the FAM83 family in pancreatic ductal adenocarcinoma (PDAC): (a) relative level of FAM83 family members in PDAC; (b) coexpression of FAM83 family members in PDAC; (c) summary of alterations in FAM83 family members in PDAC; (d) neighbor gene network of FAM83 family members.

The relative expression levels of FAM83 family members were compared in PDAC. The results showed that the expression of FAM83E was the highest while the expression of FAM83C was the lowest (Figure 2(a)). We next investigated the potential coexpression of all FAM83 family members. The Spearman correlation analysis indicated a low to moderate positive correlation among FAM83B, FAM83E, FAM83G, and FAM83H (Figure 2(b)). Further, we analyzed the molecular characteristics of all FAM83 family members and provisional dataset of TCGA was used to investigate the genetic alterations by using the cBioPortal database. Differential degrees of genetic variation among FAM83 family members are shown in Figure 2(c). FAM83H displayed the highest alteration rate (18%) of genetic variations. The alteration rates of FAM83A, FAM83B, FAM83C, FAM83D, FAM83E, FAM83F, and FAM83G were 17%, 6%, 5%, 2.4%, 8%, 5%, and 6% in the queried PDAC samples, respectively. Using GeneMANIA tools, we analyzed the relationship of FAM83 family members and constructed a network map at the gene level (Figure 2(d)). The 8 central nodes representing FAM83 family members were surrounded by 20 nodes which represent genes closely correlated with the family. The connection between FAM83C and FAM83H, FAM83C, and FAM83G, as well as FAM83H and FAM83G, was identified.

### 3.3. The Association between FAM83 Family and Tumor Pathological Stage and Patient Survival in PDAC

**Figure 3:**
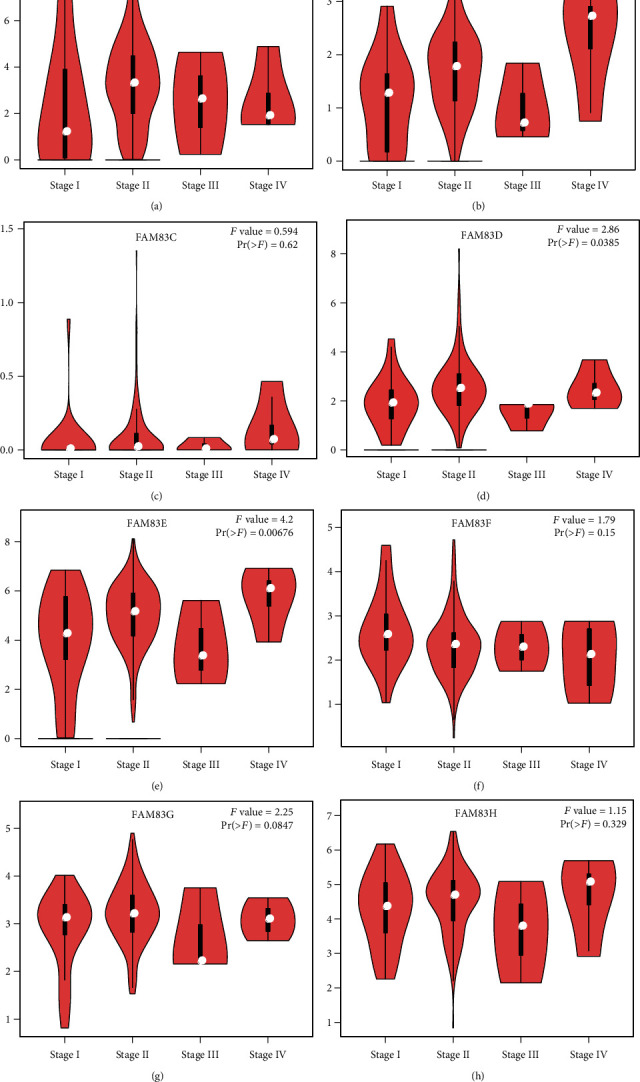
Correlation between FAM83 family members and tumor pathological stage of pancreatic ductal adenocarcinoma (PDAC) patients.

**Figure 4:**
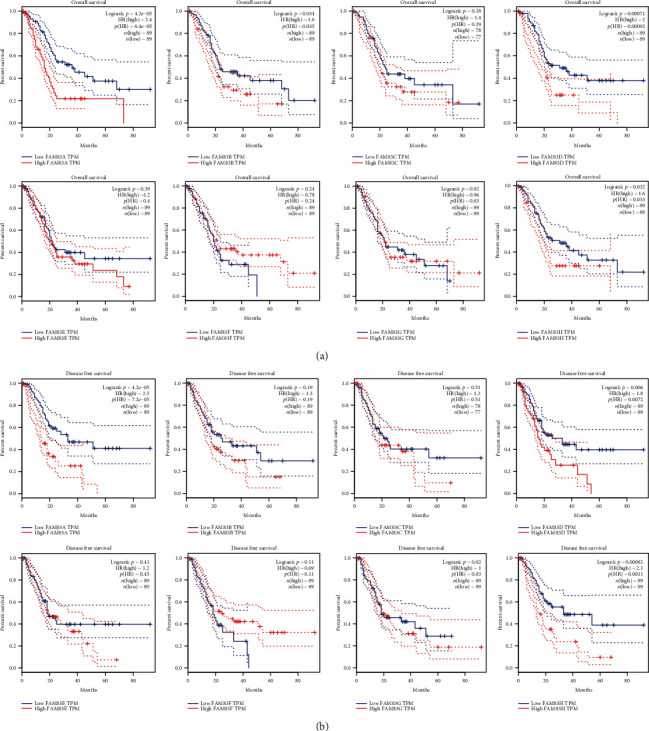
Prognostic value of FAM83 family members in pancreatic ductal adenocarcinoma (PDAC) patients in the overall survival curve (a) and disease-free survival curve (b).

To evaluate the prognostic value of FAM83 family members in PDAC, we interrogated GEPIA dataset to determine whether FAM83 family expression was associated with tumor pathological stage or patient survival. We found that higher expression of FAM83B (*p* = 0.00333), FAM83D (*p* = 0.0385), and FAM83E (*p* = 0.00676) was correlated with more advanced pathological stage (Figure 3). In contrast, no association was shown between other FAM83 family members and tumor stage. By performing Kaplan-Meier analysis, we found that higher expression levels of FAM83A (HR = 2.4, logrank *p* value = 4.2*E*-05), FAM83B (HR = 1.6, logrank *p* value = 0.034), FAM83D (HR = 2, logrank *p* value = 7.1*E*-04), and FAM83H (HR = 1.6, logrank *p* value = 0.032) were significantly associated with worse OS (Figure 4(a)). Besides, higher expression of FAM83A (HR = 2.5, logrank *p* value = 4.2*E*-05), FAM83D (HR = 1.8, logrank *p* value = 0.006), and FAM83H (HR = 2.1, logrank *p* value = 8.2*E*-04) was dramatically correlated with worse DFS (Figure 4(b)). Taken together, these data confirm the prognostic value of FAM83A, FAM83B, FAM83D, FAM83E, and FAM83H in PDAC, which may predict tumor stage or patient survival.

### 3.4. Functional and Pathway Enrichment Analyses of DEGs in FAM83 Family

**Figure 5:**
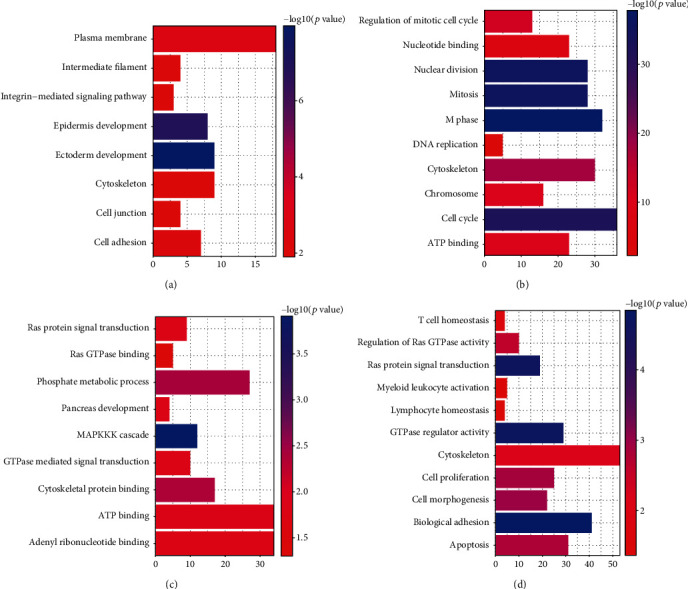
Gene Ontology (GO) enrichment analysis of differentially expressed FAM83 family members and neighboring genes in pancreatic ductal adenocarcinoma (PDAC): (a) GO enrichment analysis of FAM83A; (b) GO enrichment analysis of FAM83D; (c) GO enrichment analysis of FAM83E; (d) GO enrichment analysis of FAM83H. Color: enriched *p* value.

We next explored the activities of DEGs (i.e., FAM83A, FAM83D, FAM83E, and FAM83H) in FAM83 family members by analyzing its potential biological pathways in PDAC. The coexpression analyses for DEGs were performed by using cBioPortal dataset (Spearman's correlated coefficient > 0.6 or <−0.6, *p* value < 0.05), and 40 coexpression genes for FAM83A, 57 coexpression genes for FAM83D, 301 coexpression genes for FAM83E, and 572 coexpression genes for FAM83H were enrolled into DAVID 6.7 and subjected to functional and pathway enrichment analyses. GO enrichment analysis showed that FAM83A may be involved in “ectoderm and epidermis development, constitution of plasma membrane, intermediate filament, cell junction and cytoskeleton, regulation of cell adhesion, and integrin-mediated signaling pathway” (Figure 5(a)). FAM83D and its neighboring genes were mainly enriched in “cell cycle (M phase), DNA replication, nuclear division and constitution of cytoskeleton, chromosome, ATP binding, and nucleotide binding” (Figure 5(b)). FAM83E may play an important role in “MAPKKK cascade, phosphate metabolic process, Cytoskeletal protein binding, ATP binding, adenyl ribonucleotide binding, GTPase mediated signal transduction, Ras protein signal transduction, Ras GTPase binding, and pancreas development” (Figure 5(c)). FAM83H may act a vital role in “biological adhesion, GTPase regulator activity, Ras protein signal transduction, cell morphogenesis, cell proliferation, apoptosis, regulation of Ras GTPase activity, T cell homeostasis, myeloid leukocyte activation, and lymphocyte homeostasis” (Figure 5(d)). In KEGG analysis (Table 2), FAM83A was found to be mainly enriched in “ECM-receptor interaction, focal adhesion, and pathways in cancer” while FAM83D may participate in “cell cycle, oocyte meiosis, and p53 signaling pathway.” FAM83E was mainly associated with “axon guidance, tight junction, glycosphingolipid biosynthesis, ErbB signaling pathway, Fc gamma R-mediated phagocytosis, VEGF signaling pathway, and adherens junction,” and FAM83H was involved in “axon guidance, cell adhesion molecules (CAMs), calcium signaling pathway, tight junction, leukocyte transendothelial migration, and pathways in cancer.” These results imply that DEGs of the FAM83 family may provide important support for tumorigenesis and progression via different signaling pathways.

### 3.5. Correlations between DEGs in FAM83 Family with Important Driver Genes of PDAC

**Figure 6:**
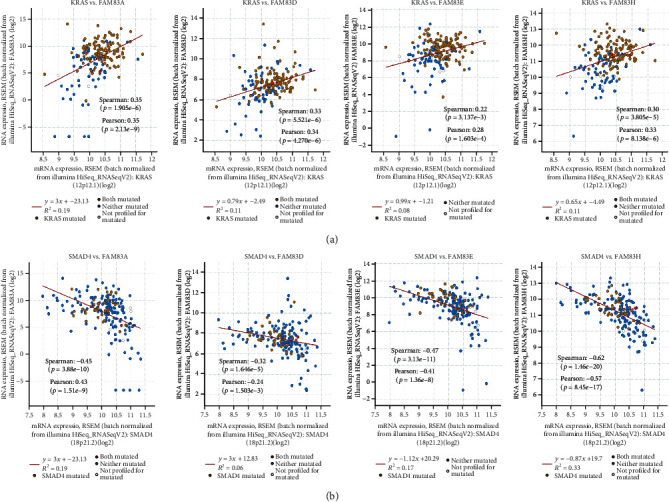
Correlation between expression levels of differentially expressed FAM83 family members with important driver genes of pancreatic ductal adenocarcinoma (PDAC) including KRAS (a) and SMAD4 (b).

We performed Spearman's correlation to explore the correlations between DEGs of FAM83 family members and important driver genes of PDAC, which are KRAS, SMAD4, TP53, and CDKN2A. As shown in Figure 6(a), KRAS expression level was positively correlated with these members (Cor = 0.35, *p* value = 1.91*E*-06 for FAM83A; Cor = 0.33, *p* value = 5.52*E*-06 for FAM83D; Cor = 0.32, *p* value = 1.28*E*-05 for FAM83E; and Cor = 0.30, *p* value = 3.81*E*-05 for FAM83H). SMAD4 expression level was negatively associated with these members (Cor = −0.45, *p* value = 3.88*E*-10 for FAM83A; Cor = −0.32, *p* value = 1.65*E*-05 for FAM83D; Cor = −0.47, *p* value = 3.13*E*-11 for FAM83E; and Cor = −0.62, *p* value = 1.46*E*-20 for FAM83H) (Figure 6(b)). However, the expression levels of TP53 and CDKN2A had no any significant correlations with FAM83 family members.

### 3.6. The Association between T Cell Infiltration and the Expression of FAM83 Family in PDAC

**Figure 7:**
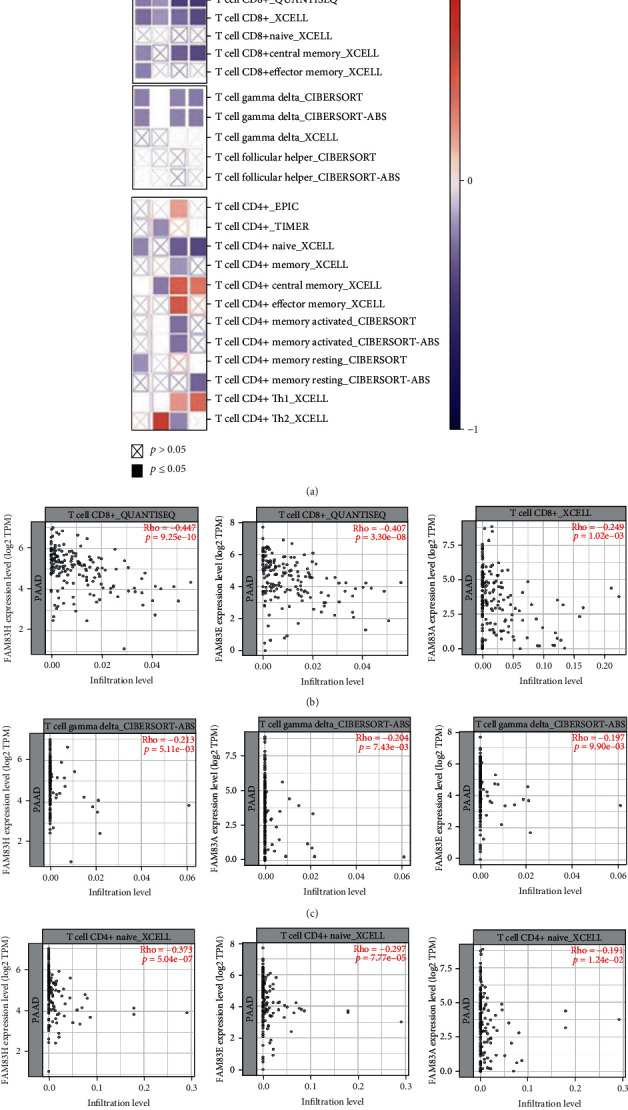
Association between the different expressions of FAM83 family with T cell infiltration levels in pancreatic ductal adenocarcinoma (PDAC): (a) R between the expression of FAM83A, D, E, and H with T cell infiltration levels including CD8+ T cell, Gamma Delta T cell, follicular helper T cell, and CD4^+^ T cell in PDAC. (b-d) Spearman's correlation between the expression of FAM83A, E, and H with CD8^+^ T cell (b), Gamma Delta T cell (c), and naive CD4^+^ T cell (d) infiltration levels. Red and blue cells showed positive and negative correlations, respectively. The intensity of color was proportional to the strength of the correlations.

Immune infiltration especially T cell infiltration is a critical factor associated with tumor progression in PDAC [26]. Therefore, we assessed the correlations of DEG expression of the FAM83 family with T cell infiltration levels including CD8^+^ T cell, Gamma Delta T cell, follicular helper T cell, and CD4^+^ T cell in PDAC by using TIMER 2.0 platforms (Figure 7(a)). After purity adjustment, the Spearman correlation analysis showed that the expression of FAM83A, FAM83D, FAM83E, and FAM83H was negatively correlated with the abundance of CD8^+^ T cell (Figure 7(b)). Negative correlations were also found between the infiltration of Gamma Delta T cell and FAM83H (Cor = −0.21, *p* value = 0.00511), FAM83A (Cor = −0.20, *p* value = 0.00743), and FAM83E (Cor = −0.20, *p* value = 0.0099) (Figure 7(c)). Besides, several kinds of CD4^+^ T cell may play more complex roles and the naive CD4^+^ T cell displayed the greatest negative correlations including FAM83H (Cor = −0.37, *p* value = 5.04*E*-07), FAM83E (Cor = −0.30, *p* value = 7.77E-05), and FAM83A (Cor = −0.19, *p* value = 0.0124) (Figure 7(d)).

### 3.7. FAM83H Was Associated with TILs and Immunomodulators in PDAC

**Figure 8:**
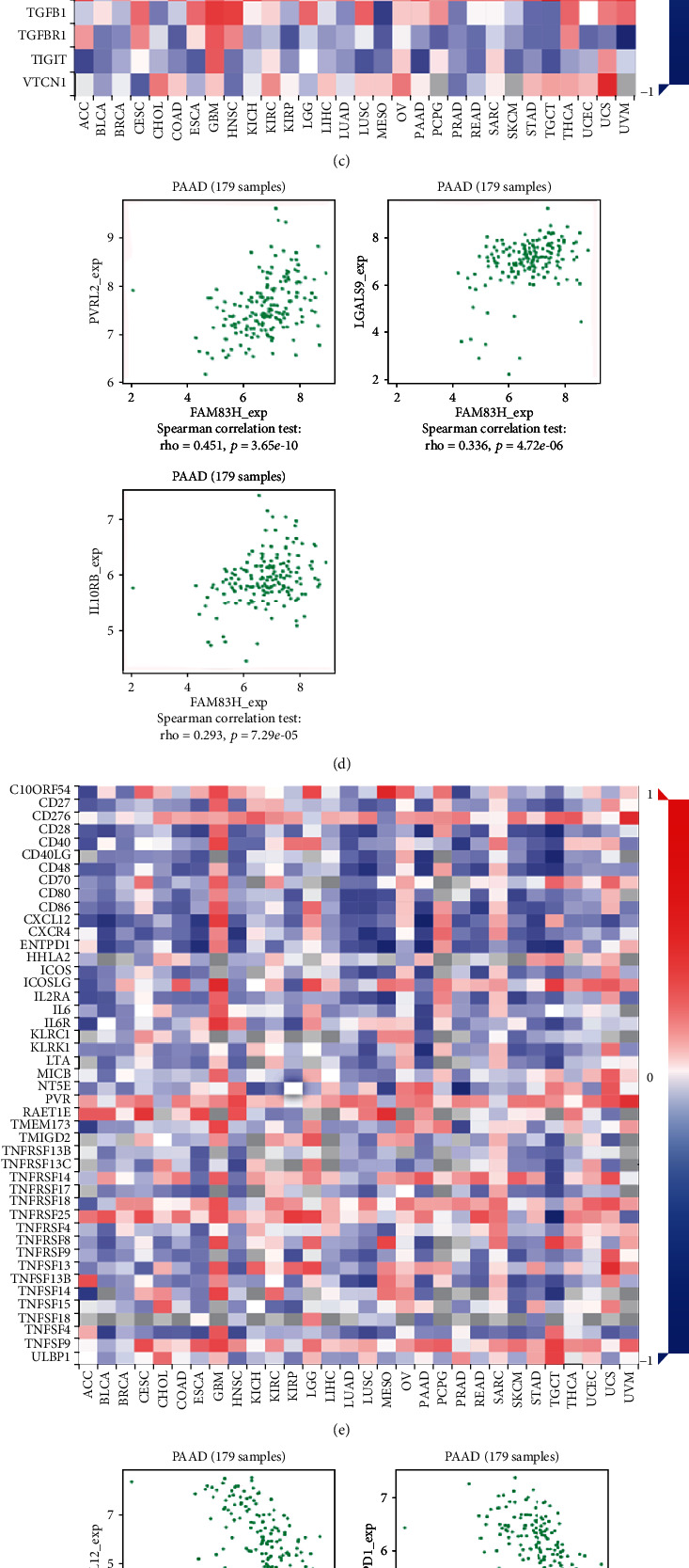
Correlations of FAM83H with tumor-infiltrating lymphocytes (TILs) and immunomodulators: (a) correlations between TILs and FAM83H expression; (B) top 4 TILs with the greatest negative Spearman correlation with FAM83H; (c) correlations between immunoinhibitors and FAM83H expression; (d) top 3 immunoinhibitors with the greatest positive Spearman correlation with FAM83H; (e) correlations between immunostimulators and FAM83H expression; (f) top 4 immunostimulators with the greatest negative Spearman correlation with FAM83H; (g) correlations between MHC molecules and FAM83H expression; (h) top 4 MHC molecules with the greatest negative Spearman correlation with FAM83H. Red and blue cells showed positive and negative correlations, respectively. The intensity of color was proportional to the strength of the correlations.

To further explore the relationship between FAM83H and immune regulation, the Spearman correlations between FAM83H with TILs and immunomodulators (including immunoinhibitors, immunostimulators, and MHC molecules) were analyzed by using the TISIDB database. As shown in Figure 8, we found that FAM83H was correlated with immune cell infiltration and immunomodulators. In detail, by analyzing different subtypes of lymphocytes, we found that FAM83H was mostly negatively correlated with infiltrating levels of effector memory CD4^+^ T cell (Cor = −0.636, *p* value < 2.2*e*-16), eosinophil (Cor = −0.6, *p* value < 2.2*e*-16), mast cell (Cor = −0.593, *p* value < 2.2*e*-16), and follicular helper T cell (Cor = −0.514, *p* value < 2.2*e*-16) (Figure 8(b)). For the immunoinhibitors, FAM83H was mostly positively correlated with PVRL2 (Cor = 0.451, *p* value = 3.7*e*-10), LGALS9 (Cor = 0.336, *p* value = 4.7*e*-06), IL10RB (Cor = 0.293, *p* value = 7.29*e*-05) (Figure 8(d)). For the immunostimulators, FAM83H was mostly negatively correlated with CXCL12 (Cor = −0.64, *p* value < 2.2*e*-16), ENTPD1 (Cor = −0.567, *p* value < 2.2*e*-16), CD28 (Cor = −0.521, *p* value < 2.2*e*-16), and KLPK1 (Cor = −0.52, *p* value < 2.2*e*-16) (Figure 8(f)). For the MHC molecules, FAM83H was mostly negatively correlated with HLA-DPA1 (Cor = −0.484, *p* value = 4.22*e*-12), HLA-DPB1 (Cor = −0.472, *p* value < 3.1*e*-11), HLA-DOA (Cor = −0.45, *p* value < 3.72*e*-10), and HLA-DRA (Cor = −0.45, *p* value < 3.88*e*-10) (Figure 8(h)). These results indicate that FAM83H may be involved in tumor specific immune response by regulating the TILs and immune molecules.

## 4. Discussion

Although FAM83 family members were proven to play a significant role in several kinds of cancer, their biological roles and prognostic value in PDAC have rarely been characterized. In the current study, we first showed that FAM83A, FAM83D, FAM83E, and FAM83H were significantly overexpressed in PDAC. And higher expression of FAM83B, FAM83D, and FAM83E was associated with tumor stage. Further, upregulation of FAM83B was significantly associated with worse OS, while upregulation of FAM83A, FAM83D, and FAM83H was correlated with both worse OS and DFS. These data suggested the predictive value of FAM83A, FAM83B, FAM83D, FAM83E, and FAM83H for the prognosis of PDAC.

FAM83 proteins are characterized by an N-terminal “domain of unknown function” called DUF1669. However, each member has a unique C terminus of variable length and their biological function and related mechanism may be distinct. By performing functional and pathway enrichment analyses, we showed that FAM83A was involved in regulation of cell adhesion, integrin-mediated signaling pathway, and ECM-receptor interaction in PDAC. Studies have proved that elevated FAM83A expression maintained essential MEK/ERK survival signaling and prevented cell death in PC cells [27]. Upregulation of FAM83A was also found in lung, ovarian, cervical, and certain brain tumor [9]. Knockdown of FAM83A increased the expression levels of *α*1, *α*3, *α*5, *β*4, and *β*5 integrins in CaSki and HeLa cells. In our study, FAM83D and its neighboring genes were mainly enriched in “cell cycle (M phase), DNA replication, nuclear division, oocyte meiosis, ATP and nucleotide binding, and p53 signaling pathway” in PDAC. In other study, exogenous FAM83D overexpression promoted, while FAM83D silencing inhibited non-small-cell lung cancer (NSCLC) cell proliferation, epithelial-mesenchymal transition, and invasion through regulating the AKT/mTOR pathway [28]. Besides, it also involved in the MEK/ERK signaling pathway and promote the entry into S phase of cell cycle in hepatocellular carcinoma [29]. These indicate that upregulation of FAM83D may enhance cancer cell division and proliferation by affecting cell cycle progression in PDAC.

Through functional enrichment analyses, we found that FAM83E and FAM83H were involved in “Ras GTPase binding, regulation of Ras GTPase activity, and Ras protein signal transduction.” As an important member of the Ras family, KRAS is a critical driver gene in PDAC, which is characterized by a nearly 100% KRAS mutation frequency. In this study, KRAS expression level was found positively correlated with FAM83A, FAM83D, FAM83E, and FAM83H. Our result also demonstrated that the expression of SMAD4, another driver gene of PDAC, was negatively associated with FAM83A, FAM83D, FAM83E, and FAM83H. Taking these together, FAM83 members may regulate or be regulated by KRAS or SMAD4 in PDAC, leading to cancer progression. It will be of great interest to investigate the detailed mechanism between FAM83 members and KARS or SMAD4 in future study.

The immune cell infiltration and tumor microenvironment have been verified to play vital roles in PDAC progression and tumor evasion [30]. In recent years, the application of immunotherapies to stimulate effector T cells to kill tumor cells has aroused great interest. However, clinical researches have shown that checkpoint inhibition therapy alone is insufficient for the treatment in PDAC [31], of which insufficient TILs are a fundamental cause of “cold” tumors and immune checkpoint unresponsiveness [32]. In our study, a significant negative correlation was suggested between the abundance of CD8^+^ T cell with FAM83A, D, E, and H in PDAC. Findings from the current research have indicated that subsets of TILs, especially CD8^+^ T cell, are strongly associated with long-term oncological outcomes in patients with PDAC and limited CD8^+^ T cell infiltration is found in most PDAC tumor centers [33, 34]. Gamma Delta T cell has been considered to play protective roles in tumorigenesis, largely on the basis of their cytotoxicity and interferon-*γ* production, which inhibits tumor progression [35]. However, the role of Gamma Delta T cell in PDAC is contradictive and it was revealed that Gamma Delta T cell may inhibit *αβ* T cell activation and promote pancreatic oncogenesis [36]. The exploration of the interaction and mechanism between Gamma Delta T cell and some potential immune regulatory factors like FAM83 family will contribute to facilitate the elucidation of the Gamma Delta T cell in PDAC formation and progression. These results indicated that the FAM83 family were not only prognostic biomarkers but also reflect some immune status of PDAC.

By assessing the correlation between FAM83H expression with tumor-infiltrating lymphocytes and immunomodulators, we found a broad correlation between FAM83H with immune cell infiltration (such as effector memory CD4^+^ T cell, eosinophil, mast cell, follicular helper T cell), immunoinhibitors (such as PVRL2, LGALS9, IL10RB), immunostimulators (such as CXCL12, ENTPD1, CD28, KLRK1), and MHC molecules (such as HLA-DPA1, HLA-DPB1, HLA-DOA, HLA-DRA). PVRL2 (CD112), known as the ligand of PVRIG and CD226, is overexpressed in different types of tumor cell and plays an immunosuppressive role in T cell function [36]. Blockade of PVRIG-PVRL2 enhanced cytotoxic function of CD8^+^ T cells, and the triple-combination blockade of PVRIG-PVRL2, TIGIT, and PD-1 resulted in the greatest increase in IFN*γ*, which means mostly enhancement of CD8^+^ effector function [37]. Besides, the enhancement of natural killer cell function was also observed by blockading the PVRL2 [38]. CD39 (ENTPD1), a molecule associated with chronic immune cell stimulation, was proven to be a marker of tumor-infiltrating CD8^+^ T cells [39]. Simoni et al. performed a transcriptomic profiling analysis and found that CD39^+^ CD8^+^ TILs were enriched in genes related to cell proliferation and exhaustion, which are characteristics of chronically stimulated T cells. At the protein level, CD39^+^ CD8^+^ TILs also showed characteristics of exhausted cells in terms of both phenotypic and functional markers in colon and lung cancers [39]. MHC-II molecules, particularly HLA-DRA, are critical for antigen presentation to CD4^+^ T cell and required for antiPD-1/PD-L1 activity in melanoma; agents that induce MHC-II positivity can be combined with PD-1/PD-L1-targeted therapy to improve response rates [40]. Thus, FAM83H, which is related to the above immune molecules, might serve as a potential immunotherapeutic target.

However, there are several limitations of our study. Although the mRNA expression of some FAM83 family members was identified as prognostic biomarkers for DFS and OS in the study, the changes of protein levels and their prognostic implication were not demonstrated. Besides, our analyses can reflect some respects of immune status in PDAC but not the global changes. Further prospective experiments and in vitro or in vivo studies are needed to validate our results and explore underlying molecular mechanisms.

In conclusion, FAM83 family members display differential degrees of overexpression and play vital roles in tumor progression and the regulation of immune cell infiltration of PDAC. Further, our study explored the correlation between DEGs in FAM83 family with tumor-infiltrating lymphocytes and highlight FAM83H as a prognostic biomarker and a potential immunotherapeutic target to treat PDAC.

## Figures and Tables

**Table 1:** mRNA levels of the FAM83 family in PDAC tissues and normal pancreas tissues at transcriptome level (Oncomine).

FAM83 family	Sample (cancer vs. normal)	Fold change	*p* value	Reference
FAM83A	12 vs. 5	2.838	7.60*E*-04	[16]
	36 vs. 16	2.681	2.41*E*-07	[17]
	11 vs. 11	2.209	0.047	[18]
	39 vs. 39	1.557	3.21*E*-05	[19]

FAM83D	36 vs. 16	4.021	9.50*E*-04	[17]
	12 vs. 5	3.399	0.010	[16]
	39 vs. 39	2.517	6.77*E*-06	[19]
	11 vs. 11	2.238	0.009	[18]

FAM83E	36 vs. 16	2.365	3.07*E*-06	[17]

FAM83H	36 vs. 16	2.972	1.16*E*-07	[17]
	11 vs. 11	2.505	0.037	[18]
	39 vs. 39	1.887	2.09*E*-06	[19]
	12 vs. 5	1.86	0.037	[16]

**Table 2:** Pathway enrichment analysis of differentially expressed genes among the FAM83 family.

KEGG pathway	Fold enrichment	*p* value	Related genes
*FAM83A*			
ECM-receptor interaction	23.28297	3.10*E*-05	LAMB3, ITGB6, ITGB4, LAMC2, ITGA3
Focal adhesion	11.67623	5.80*E*-05	LAMB3, ITGB6, ITGB4, LAMC2, ITGA3, FLNB
Pathways in cancer	4.770169	0.037837	LAMB3, SLC2A1, LAMC2, ITGA3
*FAM83D*			
Cell cycle	26.15143	1.24*E*-10	CCNB1, CDK1, CCNB2, PLK1, BUB1, BUB1B, TTK, CDC25C, CCNA2
Oocyte meiosis	23.11364	1.36*E*-07	CCNB1, CDK1, CCNB2, PLK1, BUB1, AURKA, CDC25C
p53 signaling pathway	21.36555	5.94*E*-04	CCNB1, CDK1, CCNB2, RRM2
*FAM83E*			
Axon guidance	4.125203	0.001289	SEMA4G, EFNA1, EFNA2, CFL2, EFNB1, MAPK3, PPP3CB, EFNA4, CXCL12
Tight junction	3.971277	0.001647	CLDN7, MPDZ, CGN, CRB3, MYH14, AMOTL1, TJP3, SRC, AKT3
Glycosphingolipid biosynthesis	9.460465	0.00795	FUT6, FUT3, B3GNT3, FUT2
ErbB signaling pathway	4.077787	0.014669	CBLC, ERBB2, MAPK3, MAPK10, SRC, AKT3
Fc gamma R-mediated phagocytosis	3.734394	0.020746	MARCKSL1, CFL2, MAPK3, VASP, AKT3, DNM2
VEGF signaling pathway	3.94186	0.036037	PLA2G10, MAPK3, PPP3CB, SRC, AKT3
Adherens junction	3.839474	0.039139	FGFR1, ERBB2, MAPK3, TCF7L2, SRC
*FAM83H*			
Axon guidance	3.742273	3.61*E*-05	NGEF, EFNB1, EPHB4, CXCL12, EPHA2, SLIT3, SEMA5A, SEMA3G, FYN, CFL2, RAC1, PPP3CB, SEMA4B, EFNA4, RHOD
Cell adhesion molecules (CAMs)	2.925777	0.002361	F11R, CLDN7, SDC1, CLDN4, CD34, NRXN3, PECAM1, NLGN4X, JAM2, SDC4, NEGR1, CDH5
Calcium signaling pathway	2.377194	0.007799	SLC8A3, EDNRB, PLCB3, SLC8A1, PTGER3, ADORA2A, TACR1, ERBB2, PDE1A, PPP3CB, PLCD3, PTGFR, ITPR1
Tight junction	2.641933	0.008055	F11R, EPB41L3, CLDN7, CTTN, CLDN4, MPDZ, CGN, MYH14, JAM2, TJP3, MYH10
Leukocyte transendothelial migration	2.727419	0.010253	F11R, CLDN7, CLDN4, PECAM1, RAC1, RAPGEF4, JAM2, CXCL12, CDH5, VASP
Pathways in cancer	1.66805	0.04474	CKS1B, FGFR1, ERBB2, STAT5B, SMAD4, ITGA3, BCL2L1, MAPK10, FZD4, JUP, CBLC, LAMB3, BCL2, RAC1, PIAS2, TRAF4, GSTP1

## Data Availability

All database including Oncomine (https://www.oncomine.org), GEPIA (http://gepia.cancer-pku.cn/index.html), cBioPortal Database (http://www.cbioportal.org/), DAVID 6.7 (https://david-d.ncifcrf.gov/), GeneMANIA database (http://genemania.org/), TIMER 2.0 (http://timer.cistrome.org/), and TISIDB (http://cis.hku.hk/TISIDB) are freely available as public resources.

## Abbreviations

PC: Pancreatic cancer
PDAC: Pancreatic ductal adenocarcinoma
FAM83: Family with sequence similarity 83
DEGs: Differentially expressed genes
GEPIA: Gene Expression Profiling Interactive Analysis
TCGA: The Cancer Genome Atlas
GTEx: The Genotype-Tissue Expression
FPKM: Fragments per kilobase per million
GO: Gene Ontology
KEGG: Kyoto Encyclopedia of Genes and Genomes
TILs: Tumor-infiltrating lymphocytes
MHC: Major histocompatibility complex.
